# Bronchiectasis-Associated Hospitalizations in Germany, 2005–2011: A Population-Based Study of Disease Burden and Trends

**DOI:** 10.1371/journal.pone.0071109

**Published:** 2013-08-01

**Authors:** Felix C. Ringshausen, Andrés de Roux, Mathias W. Pletz, Nina Hämäläinen, Tobias Welte, Jessica Rademacher

**Affiliations:** 1 Department of Respiratory Medicine, Hannover Medical School, Hannover, Germany; 2 Center for Respiratory Medicine at the Charlottenburg Castle, Berlin, Germany; 3 Center for Infectious Diseases and Infection Control, Jena University Hospital, Jena, Germany; 4 Institute for Lung Research, Berlin, Germany; National Institute of Environmental Health Sciences, United States of America

## Abstract

**Background:**

Representative population-based data on the epidemiology of bronchiectasis in Europe are limited. The aim of the present study was to investigate the current burden and the trends of bronchiectasis-associated hospitalizations and associated conditions in Germany in order to inform focused patient care and to facilitate the allocation of healthcare resources.

**Methods:**

The nationwide diagnosis-related groups hospital statistics for the years 2005–2011 were used in order to identify hospitalizations with bronchiectasis as any hospital discharge diagnosis according to the *International Classification of Diseases, 10th revision*, code J47, (acquired) bronchiectasis. Poisson log-linear regression analysis was used to assess the significance of trends. In addition, the overall length of hospital stay (LOS) and the in-hospital mortality in comparison to the nationwide overall mortality due to bronchiectasis as the primary diagnosis was assessed.

**Results:**

Overall, 61,838 records with bronchiectasis were extracted from more than 125 million hospitalizations. The average annual age-adjusted rate for bronchiectasis as any diagnosis was 9.4 hospitalizations per 100,000 population. Hospitalization rates increased significantly during the study period, with the highest rate of 39.4 hospitalizations per 100,000 population among men aged 75–84 years and the most pronounced average annual increases among females. Besides numerous bronchiectasis-associated conditions, chronic obstructive pulmonary disease (COPD) was most frequently found in up to 39.2% of hospitalizations with bronchiectasis as the primary diagnosis. The mean LOS was comparable to that for COPD. Overall, only 40% of bronchiectasis-associated deaths occurred inside the hospital.

**Conclusions:**

The present study provides evidence of a changing epidemiology and a steadily increasing prevalence of bronchiectasis-associated hospitalizations. Moreover, it confirms the diversity of bronchiectasis-associated conditions and the possible association between bronchiectasis and COPD. As the major burden of disease may be managed out-of-hospital, prospective patient registries are needed to establish the exact prevalence of bronchiectasis according to the specific underlying condition.

## Introduction

Bronchiectasis is a chronic and etiologically heterogeneous disease, which is commonly characterized by a vicious circle of impaired mucociliary clearance, bronchial infection and inflammation, resulting in a structural airway damage with abnormal and permanent widening [1]. So far, only limited data regarding the epidemiology of bronchiectasis in the United States (US), Asia, New Zealand and Finland are available, whereas representative population-based data for Central Europe, particularly for Germany, are virtually unavailable [2]–[7]. In large part, the existing epidemiological data have been derived from hospitalization, billing of health services and health insurance data [2], [5], [8], [9].

There is growing evidence that, due to antimicrobial treatment and immunization as well as the increasing global burden of chronic obstructive pulmonary disease (COPD), the underlying etiologies and with them the epidemiology of bronchiectasis is changing [2], [10], [11]. While traditionally, children and young adults with congenital or postinfectious conditions (e.g. cystic fibrosis (CF), primary ciliary dyskinesia (PCD) and whooping cough, measles and tuberculosis (TB), respectively) have been considered as the predominant bronchiectasis patient population, adults aged ≥60 years with a substantial proportion of never-smoking women and subjects with COPD are increasingly reported in the recent literature [7], [12], [13].

In Germany, population-based data regarding hospitalizations are available at a federal level. Although the *International Classification of Diseases, 10^th^ revision* (ICD-10) diagnosis codes are used principally for the billing of health services purposes, they commonly serve as a basis in epidemiological research [2], [8], [14]. Epidemiological data and health services research on bronchiectasis are essential in order to inform specialized and tailored patient care according to these patients’ specific needs and to facilitate the reasonable allocation of healthcare resources. The aim of the present study was to provide insights into the current burden and the trends of bronchiectasis-associated hospitalizations in Germany with regard to age- and sex-specific as well as age-adjusted hospitalization rates, putative complications, common comorbidities, associated etiologies, overall length of hospitalization and mortality.

## Methods

### Ethics Statement

Because this study is based on anonymous and publicly accessible routine data, institutional review board approval and patient consent was not required.

### Data Sources and Study Population

The present study is based on ICD-10 code J47, (acquired) bronchiectasis. We extracted data from the official nationwide German diagnosis-related groups (DRG) hospital statistics, which are publicly accessible and provided by the German Federal Statistical Office [15] in collaboration with German Federal Health Monitoring Information System [16]. All German hospitals using DRG billing of medical services are legally obligated to transmit these data in response to an annual written survey. Consequently, hospitals for prevention, rehabilitation, mental and mood disorders as well as day care units were not included in our analysis. In the year 2000, the ICD-9 was replaced by the current classification in Germany. Since the year 2005, all associated secondary ICD-10 hospital discharge codes are additionally transmitted to the Federal Statistical Office.

In general, primary ICD-10 diagnosis codes are regarded as the principal condition identified during hospitalization, while secondary codes indicate associated or contributing conditions (comorbidities and/or complications). For clarity, associated primary and secondary diagnoses were classified in putative bronchiectasis-associated complications, bronchiectasis-unrelated comorbidities and diagnoses commonly associated with, but not necessarily being the etiology of bronchiectasis [17], [18].

Depersonalized DRG diagnosis data were provided for the whole of Germany as absolute numbers stratified by age groups (in 5-year intervals), sex and year of diagnosis. Accordingly, data on associated total hospital costs were not available for analysis. Additional variables included associated primary and secondary conditions as indicated by three-digit ICD-10 codes, overall length of hospital stay (LOS) as well as nationwide and in-hospital mortality for bronchiectasis as the primary diagnosis. For comparison, data regarding the overall LOS were analyzed for all hospitalizations regardless of the primary ICD-10 code as well as ICD-10 code J44 (other chronic obstructive pulmonary disease). Data about bronchiectasis as a primary cause of death are based on the official causes of death statistics from the German Federal Statistical Office and were provided as absolute numbers stratified by age groups (in 5-year intervals), sex and year of diagnosis [15]. The data are acquired within an annual census from mandatory death certificates and statistical bulletins of mortality using the World Health Organization edition of the ICD-10.

### Data and Statistical Analysis

Analysis comprised all records with bronchiectasis as the primary hospital discharge diagnosis from 2005 through 2011. Moreover, a second analysis included bronchiectasis as either a primary or a secondary diagnosis in the same period. Official German census age- and sex-specific population data were used as the denominator for all calculations [15]. Age adjustment was performed by the direct method in order to control for different age distributions across Germany and to allow for comparison between different years. Age-adjusted hospitalization rates were calculated using the latest available German Census Standard Population as the reference population [15].

Poisson log-linear regression analysis was used to assess the significance of trends. Standard errors were scaled using Pearson’s chi-square statistics in order to account for overdispersion. Continuous data were checked for normal distribution using the Kolmogorov-Smirnov test before calculating means. P-values and 95% confidence intervals (CI) were calculated from Wald statistics and bootstrapping, respectively, with statistical significance set to p<0.05. Accordingly, differences were considered statistically significant if 95% CIs were not overlapping. In addition, the annual percentage change (APC) was calculated and associated diagnoses were analyzed for two cohorts: (1) bronchiectasis as the primary diagnosis and (2) bronchiectasis as either a primary or a secondary diagnosis between 2005 and 2011, each. Moreover, the APC of the rate of associated primary and secondary diagnoses per 1,000 hospitalizations with any diagnosis of bronchiectasis was calculated. Data analysis was performed using IBM SPSS Statistics, version 20 (IBM Corp., New York, NY).

## Results

### Hospitalizations and Hospitals

Between 2005 and 2011, the average annual German population was 82.1 million, ranging from 82.4 million in 2005 to 81.1 million in 2011. There were on average 17.9 (95% CI 17.4–18.3) million hospitalizations per year, which were steadily increasing from 17.0 to 18.8 million. On average, 1,673 (95% CI 1,633–1,719) hospitals were subject to DRG billing of medical services, with a continuous downward trend from 1,770 hospitals in 2005 to 1,601 hospitals in 2011. In this period, the average overall number of hospitals or hospital-like healthcare facilities in Germany was 2,087 (95% CI 2,067–2,108). Correspondingly, approximately 80% of all hospitals were included in our analysis.

### Burden of Bronchiectasis as a Hospital Discharge Diagnosis

**Figure 1:**
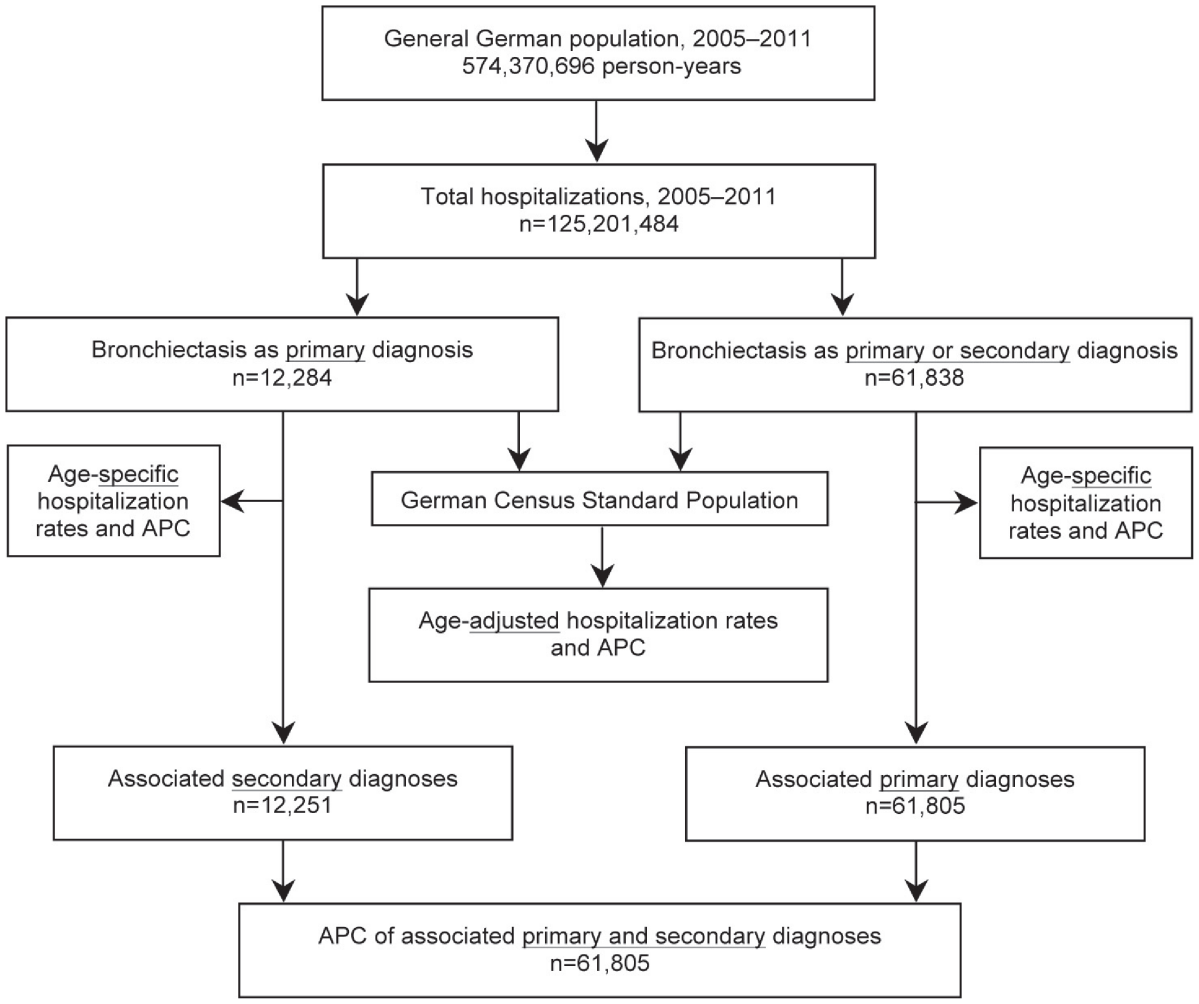
Data flow diagram. APC: annual percentage change.

**Figure 2:**
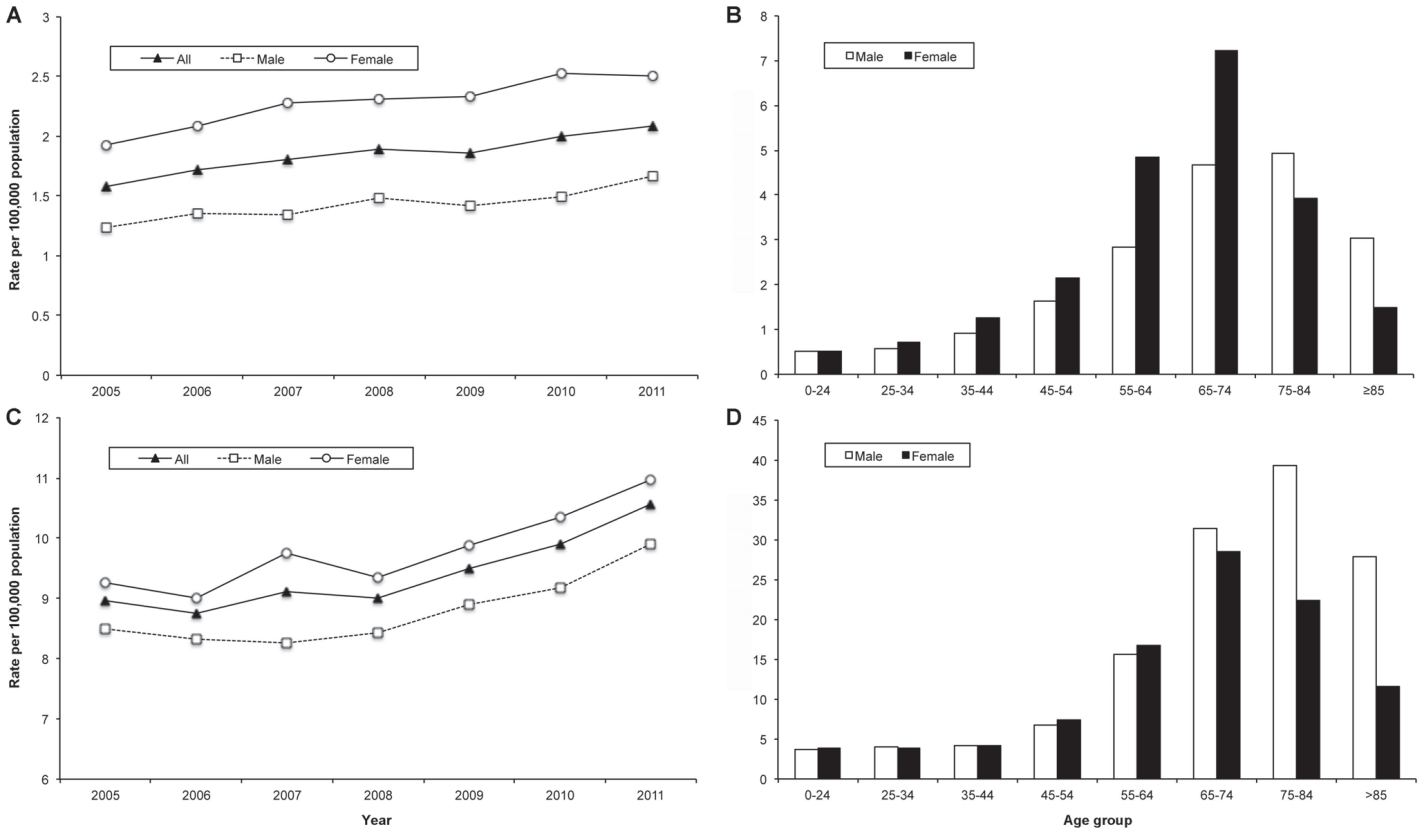
Annual age-adjusted and average annual age-specific hospitalization rates. (A) Annual age-adjusted hospitalization rate and (B) average annual age-specific hospitalization rate of bronchiectasis as the primary hospital discharge diagnosis by year, age group and sex, respectively; (C) annual age-adjusted hospitalization rate and (D) average annual age-specific hospitalization rate of bronchiectasis as any diagnoses by year, age group and sex, respectively, Germany, 2005–2011.

From 2005 to 2011, a total of 125.2 million hospitalizations were analyzed over an observational period of 574.4 million person-years. In total, 12,284 hospitalizations with bronchiectasis as the primary hospital discharge diagnosis were identified (Figure 1), of which 7,329 (60%) were among females and 5,839 (48%) were among subjects aged ≥65 years. The average annual age-adjusted rate of hospitalizations with bronchiectasis as the primary diagnosis was 1.8 (95% CI 1.7–2.0) hospitalizations per 100,000 population. This rate was significantly higher among females compared to males (2.3 (95% CI 2.1–2.4) vs. 1.4 (95% CI 1.3–1.5) per 100,000 population) and showed further variation depending on age, with the highest age-specific rate of 7.2 hospitalizations per 100,000 population among women aged 65 to 74 years (Figure 2).

Overall, 61,838 hospitalizations with bronchiectasis as either a primary or a secondary hospital discharge diagnosis were identified (Figure 1), of which 30,971 (50%) were among females and 32,637 (53%) were among subjects aged ≥65 years. The average annual age-adjusted rate of any bronchiectasis-associated hospitalizations was 9.4 (95% CI 9.0–9.9) per 100,000 population. Again, this rate was significantly higher among females compared to males (9.8 (95% CI 9.3–10.3) vs. 8.8 (95% CI 8.4–9.2) per 100,000 population). However, there was considerable variation with age, with the highest age-specific rate of 39.4 hospitalizations per 100,000 population among men aged 75 to 84 years (Figure 2).

### Trends of Bronchiectasis as a Hospital Discharge Diagnosis

Between 2005 and 2011, the number of hospitalization for bronchiectasis as the primary diagnosis per year steadily increased from 1,449 to 2,009. Likewise, the number of hospitalizations for bronchiectasis as any diagnosis per year steadily increased from 8,152 to 10,133. According to this, the annual proportion of all bronchiectasis-associated hospitalizations among the overall number of hospitalizations significantly increased from 0.048% to 0.054%, with an average annual increase of 2.0% (95% CI 1.0–3.6; *P* = 0.0001). Figure 2 shows that the age-adjusted rates of hospitalizations increased for both bronchiectasis as the primary diagnosis and bronchiectasis as a primary or a secondary diagnosis. The overall age-adjusted rate of all bronchiectasis-associated hospitalizations increased significantly from 8.9 to 10.6 per 100,000 population, with an average increase of 2.9% (95% CI 1.7–4.2; p<0.00001) per year. Table 1 shows the average annual changes stratified by sex, age group and bronchiectasis diagnosis. Among males, the annual age-adjusted hospitalization rate increased from 8.5 to 9.9 per 100,000 population. This upward trend was even more pronounced among females, with an increase of 9.2 to 11.0 per 100,000 population (Figure 2), with an APC of the age adjusted hospitalisation rate of 4.3% (3.0–5.6; *P*<0.00001; Table 1).

**Table 1:** Average annual percentage change of bronchiectasis as a hospital discharge diagnosis, stratified by sex, bronchiectasis diagnosis and age group.

	Males			Females		
Age group (years)	Annual % change	95% CI (Wald)	*P* value	Annual % change	95% CI (Wald)	*P* value
**Bronchiectasis as the primary hospital discharge diagnosis (n = 12,284)**						
0–24	1.7	−3.0–6.5	0.48	6.2	−0.2–12.6	0.056
25–34	8.3	1.3–15.8	0.020	4.2	−3.2–11.6	0.27
35–44	2.7	0.1–5.5	0.046	3.0	−0.6–6.5	0.105
45–54	6.2	2.0–10.6	0.003	0.2	−2.7–3.1	0.91
55–64	−1.5	−4.4–1.4	0.31	−1.5	−5.0–1.9	0.39
65–74	6.9	2.9–11.0	0.0006	10.3	7.1–13.7	<0.000001
75–84	8.5	4.1–13.0	<0.0001	5.6	−0.3–11.5	0.064
≥85	−0.1	−7.1–6.9	0.97	5.8	−5.4–16.9	0.31
**All ages**	**4.2**	**2.7–5.7**	**<0.000001**	**4.3**	**3.0–5.6**	**<0.000001**
**Bronchiectasis as a primary or secondary hospital discharge diagnosis (n = 61,838)**						
0–24	0.9	−3.1–4.9	0.67	1.2	−0.9–3.3	0.27
25–34	1.7	−1.2–4.6	0.24	7.4	3.7–11.3	0.00007
35–44	4.5	2.4–6.7	0.00003	1.6	−1.7–4.9	0.35
45–54	4.4	1.6–7.2	0.002	−0.1	−1.8–1.7	0.95
55–64	0.3	−2.8–3.4	0.84	−2.2	−4.0–(−0.4)	0.014
65–74	5.8	4.6–6.9	<0.000001	6.5	4.7–8.3	<0.000001
75–84	1.6	0.3–2.9	0.014	5.6	3.4–7.9	0.000001
≥85	4.2	2.1–6.4	0.00008	2.9	−2.3–8.0	0.28
**All ages**	**2.7**	**1.3–4.2**	**0.0002**	**3.0**	**1.7–4.2**	**0.000005**

### Burden and Trends of Associated Primary and Secondary Diagnoses

**Figure 3:**
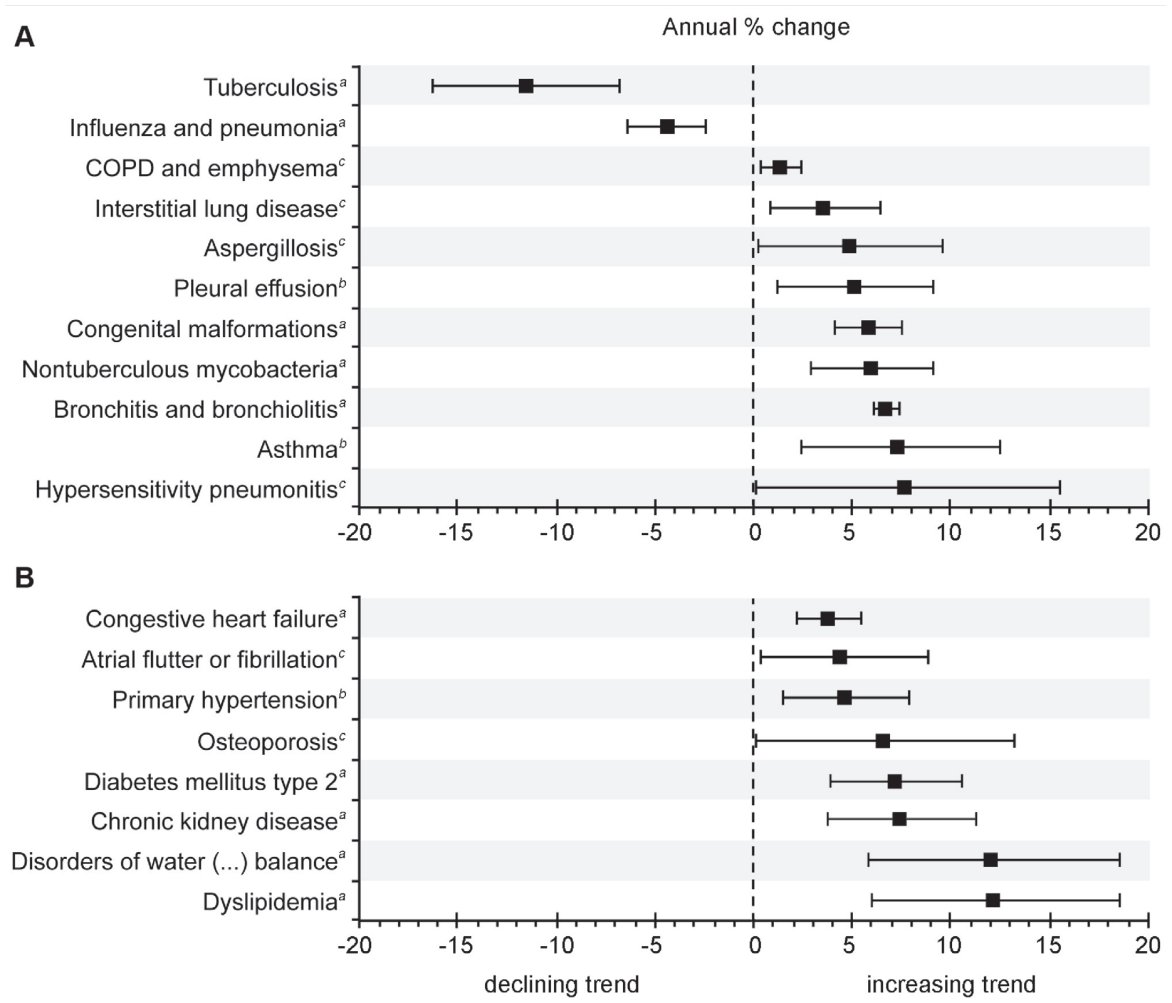
Average annual percentage change of the rate of associated primary and secondary diagnoses. Average annual percentage change of the rate of associated primary and secondary diagnoses per 1,000 hospitalizations with any diagnosis of bronchiectasis, Germany, 2005–2011, among (A) diagnoses commonly associated with bronchiectasis and (B) bronchiectasis-unrelated comorbidities. Non-significant trends are not shown. Bars indicate 95% confidence intervals calculated from Poisson log-linear regression analysis (Wald statistics). *^a^*Statistical significance at *P*<0.001. *^b^*Statistical significance at *P*<0.01. *^c^*Statistical significance at *P*<0.05.

Secondary ICD-10 codes were available for analysis from 12,251 of 12,284 subjects (99.7%) with a primary diagnosis of bronchiectasis (Figure 1). Table 2 lists conditions, which are commonly considered associated with and/or predisposing to bronchiectasis, while Table 3 shows the ten most frequent bronchiectasis-unrelated comorbidities. Moreover, this permitted the reverse analysis of associated primary diagnoses when bronchiectasis was a secondary diagnosis (Table 4). Figure 3 shows the analysis of the average annual change of the overall rate of associated primary and secondary diagnoses per 1,000 hospitalizations with bronchiectasis. The average number of secondary diagnoses per primary diagnosis of bronchiectasis was 4.6 (95% CI 4.4–4.9), with an average annual increase of 3.6% (95% CI 2.7–4.5; *P*<0.00001), ranging from 4.2 in 2005 to 5.2 in 2011.

**Table 2:** Secondary diagnoses when bronchiectasis is the primary diagnosis during hospitalization, classified by putative infectious and non-infectious complications as well as diagnoses commonly associated with bronchiectasis (n = 12,251).

ICD-10	Secondary diagnosis			Frequency	Percentage
**Putative infectious and non-infectious bronchiectasis-associated complications**					
J96		Respiratory failure	2,831		23.1
J20–J22, J40–J42		Bronchitis and bronchiolitis	1,955		16.0
R04		Hemoptysis	1,831		14.9
J09–J18		Influenza and pneumonia	1,074		8.8
I27		Pulmonary heart diseases	598		4.8
R64		Cachexia	358		2.9
**Diagnoses commonly associated with bronchiectasis**					
J43–J44		COPD and emphysema		4,799	39.2
J45–J46		Asthma		787	6.4
J32		Chronic sinusitis		461	3.8
K21		Gastroesophageal reflux disease		412	3.4
J84		Interstitial lung disease		336	2.7
D38		Airway or chest neoplasm of uncertain or unknown behavior		320	2.6
M05–M06		Rheumatoid arthritis		254	2.1
D80–D84		Primary immunodeficiencies		247	2.0
K50–K52		Inflammatory bowel diseases		167	1.4
B44		Aspergillosis		130	1.1
M30–M36		Systemic connective tissue diseases		94	0.8
Q30–Q34		Congenital malformations of the respiratory system^a^		89	0.7
D86		Sarcoidosis		92	0.8
A15–A19		Tuberculosis		62	0.5
J60–J65		Pneumoconioses		48	0.4
A31		Nontuberculous mycobacteria		54	0.4
E84		Cystic Fibrosis		34	0.3

a Including primary ciliary dyskinesia. COPD: chronic obstructive pulmonary disease; ICD-10: *International Classification of Diseases, 10th revision*.

**Table 3:** Most frequent bronchiectasis-unrelated comorbidities among secondary diagnoses when bronchiectasis is the primary hospital diagnosis (n = 12,251).

ICD-10	Secondary diagnosis	Frequency	Percentage
I10	Primary hypertension	3,470	28.3
E11	Diabetes mellitus type 2	1,080	8.8
I25	Coronary artery disease	1,047	8.5
M81	Osteoporosis without pathological fracture	771	6.3
I50	Congestive heart failure	741	6.0
E87	Disorders of water, electrolyte, and acid-base balance	711	5.8
I48	Atrial flutter or fibrillation	697	5.7
E78	Disorders of lipoprotein metabolism and other lipidemias	635	5.2
N18	Chronic kidney disease	549	4.5
E66	Obesity	494	4.0

ICD-10: International Classification of Diseases, 10th revision.

**Table 4:** Primary diagnoses among hospitalizations with bronchiectasis as any diagnosis, classified by putative infectious and non-infectious complications as well as diagnoses commonly associated with bronchiectasis.

ICD-10	Primary diagnosis	Frequency	Percentage
J47	Bronchiectasis	12,251	19.8
**Putative infectious and non-infectious bronchiectasis-associated complications**			
J09–J18	Influenza and pneumonia	7,174	11.6
J20–J22, J40–J42	Bronchitis and bronchiolitis	2,410	3.9
J96	Respiratory failure	1,491	2.4
R04	Hemoptysis	547	0.9
I27	Pulmonary heart disease	281	0.5
**Diagnoses commonly associated with bronchiectasis**			
J43, J44	COPD and emphysema	12,240	19.8
E84	Cystic fibrosis	4,456	7.2
J84	Interstitial lung disease	1,852	3.0
C34	Lung cancer	1,304	2.1
J45–J46	Asthma	823	1.3
D38	Airway or chest neoplasm of uncertain or unknown behavior	516	0.8
D80–D84	Primary immunodeficiencies	478	0.8
B44	Aspergillosis	472	0.8
M30–M36	Systemic connective tissue diseases	335	0.5
A31	Nontuberculous mycobacteria	252	0.4
A15–A19	Tuberculosis	230	0.4
D86	Sarcoidosis	235	0.4
M05–M06	Rheumatoid arthritis	223	0.4
Q30–Q34	Congenital malformations of the respiratory system^a^	130	0.2
J32	Chronic sinusitis	112	0.2
J67	Hypersensitivity pneumonitis	125	0.2
J69	Aspiration pneumonia	112	0.2
K50–K52	Inflammatory bowel diseases	110	0.2
	Other primary diagnosis	13,646	22.1
**Total**		**61,805**	**100**

a Including primary ciliary dyskinesia. COPD: chronic obstructive pulmonary disease; ICD-10: *International Classification of Diseases,10th revision.*

Table 2 shows that COPD and emphysema (ICD-10 codes J43 and J44, analyzed as one entity) were the predominant secondary diagnosis in 39.2% of hospitalizations with bronchiectasis as the primary diagnosis (n = 4,799). Among all records with bronchiectasis as any hospital discharge diagnosis, bronchiectasis itself was the most frequent primary diagnosis (n = 12,251; 20%), followed by COPD and emphysema (n = 12,240; 20%), influenza and pneumonia (ICD-10 codes J09–J18, summarized as one entity; n = 7,174; 12%), CF (n = 4,456; 7%) and a variety of other primary diagnoses (Table 4).

Accordingly, COPD and emphysema as well as influenza and pneumonia had by far the highest overall rates of 267 and 133 associated primary or secondary diagnoses per 1,000 hospitalized patients with any diagnosis of bronchiectasis, respectively. Figure 3 shows the trends of the rates of bronchiectasis-associated conditions with significant average annual changes. Remarkably, TB as well as influenza and pneumonia were the only associated diagnoses showing a significantly declining trend (*P*<0.0001), while the trend of associated nontuberculous mycobacterial (NTM) infections significantly increased during the study period (*P*<0.001; Figure 3).

Bacteria as causative agents were recorded for 4,149 of 12,251 subjects (34%) with a primary diagnosis of bronchiectasis: 989 subjects had ICD-10 code B95, streptococci and staphylococci, while 3,160 subjects had ICD-10 code B96, miscellaneous specified bacteria (including Haemophilus spp., Moraxella spp., Enterobacteriaceae, Pseudomonas spp. and other nonfermenters). Between 2005 and 2011, a significantly increasing trend was observed for bacteria as causative agents as secondary diagnoses (ICD-10 B95: APC 14.3% (95% CI 10.5–18.2); *P*<0.00001; B96: APC 11.0% (95% CI 8.3–13.8; *P*<0.00001)).

### Length of Hospital Stay and Mortality

The mean overall LOS for bronchiectasis as the primary diagnosis of 10.1 (95% CI 9.8–10.5) days was comparable to the LOS for COPD as the primary diagnosis (ICD-10 code J44; 10.3 (95% CI 10.0–10.7) days). However, it was significantly longer than the LOS for all hospitalizations (all ICD-10 codes; mean 8.1 (95% CI 7.9–8.4) days). The mean overall LOS did not differ by gender for bronchiectasis (males vs. females; 10.0 (95% CI 9.7–10.2) vs. 10.2 (95% CI 9.8–10.7) days) as well as for all hospitalizations (males vs. females; 8.1 (95% CI 7.9–8.3) vs. 8.2 (95% CI 8.0–8.5) days).

According to the causes in German death statistics, there were a total of 164 notified deaths due to bronchiectasis among 93 males and 71 females. Correspondingly, the overall age-adjusted mortality rate of 0.03 per 100,000 population was low. Of those deceased, 131 subjects (80%) were aged ≥65 years and 80 (49%) were aged ≥75 years. Notably, when comparing these numbers to the 66 deaths among 12,284 hospitalizations with a primary diagnosis of bronchiectasis (overall crude in-hospital mortality 0.5%; 36 males, 30 females), it became evident that only 40% of all deaths (66 of 164), 37% of deaths among those aged ≥65 years (48 of 131) and even 30% of deaths among those aged ≥75 years (24 of 80) occurred inside the hospital. Overall, mortality did not change significantly between 2005 and 2011 (data not shown).

## Discussion

The present study provides evidence for changing trends in the epidemiology of bronchiectasis-associated hospitalizations, with COPD as the most frequent associated condition. Overall, hospitalization rates increased during the study period, with the highest average annual increase among females with bronchiectasis as the primary diagnosis. Beyond, our results confirm the diversity of bronchiectasis-associated conditions and may be relevant to the focused care of bronchiectasis patients and the future allocation of healthcare resources.

Population-based data on bronchiectasis-associated hospitalizations are limited [2], [3], [5], [8]. We found an overall age-adjusted hospitalization rate for any diagnosis of bronchiectasis of 9.4 per 100,000 population, which is somewhat less than the rates of 16.4 and 16.5 per 100,000 population from the Hong Kong Government statistics for the year 1990 and a recent study from the US for the period from 1993 to 2006, respectively [3], [8]. In agreement with the later study by Seitz and co-workers, our average annual age-adjusted hospitalization rate for bronchiectasis as the primary diagnosis was 1.8 per 100,000 population, with an increasing trend that was most pronounced among females with bronchiectasis as the primary diagnosis [8]. Hospitalization rates were generally higher among aged subjects, as reported previously [2], [5], [6], [8].

In line with the steady downtrend of TB in the general German population and the conclusion of a Finnish study, which analyzed bronchiectasis between 1972 and 1992 according to ICD-8 and ICD-9 hospital discharge diagnosis codes, we observed a significant decline of TB as well as influenza and pneumonia among patients with bronchiectasis [2], [19]. However, in contrast to our findings, the Finnish study described a significantly declining trend of bronchiectasis-associated hospitalizations. However, this study was performed before the widespread use of high resolution computed tomography (HRCT) of the chest as the gold standard for the confirmation of bronchiectasis [2].

Altogether, the upward trend of hospitalization rates which we observed does not necessarily imply that the true prevalence of bronchiectasis-associated hospitalizations is increasing, but may rather indicate the increasing routine use of HRCT and, hence, the growing recognition of bronchiectasis [9]. The increasing number of most bronchiectasis-unrelated comorbidities, along with the upward trend regarding the average number of secondary diagnoses per primary diagnosis of bronchiectasis, may reflect the increasingly aged and comorbid population during the study period. In our study, the proportion of females among hospitalizations for bronchiectasis as the primary diagnosis of 60% was comparable to previous studies, whereas it was only 50% among all hospitalizations for bronchiectasis [2], [5], [8], [9]. In contrast to our results, previous studies found that hospitalization rates were continuously increasing with age and were highest among subjects within the very advanced age groups [5], [8]. Thus, these findings may reflect age- and sex-related differences regarding the access to and the utilization of health services in Germany. Between 2005 and 2011, on average 48% of the total male, but only 43% of the total female German population aged ≥65 years (and 60% vs. 54% among those aged ≥75 years) had been hospitalized (data not shown), despite a clear predominance of females in this subpopulation [15]. This suggests that, although the most pronounced increases of hospitalization rates were observed among females, bronchiectasis may still be underdiagnosed among the more advanced age groups in Germany, particularly among elderly women [6]. On the one hand, this argues against more severe disease and frequent readmissions among the more advanced age groups.

Our findings have several important implications. Although we were unable to calculate the exact associated costs for the in-patient care of bronchiectasis patients, our data on the mean LOS emphasize that bronchiectasis may account for a significant, though underappreciated economic burden in healthcare [5], [8]. Moreover, our results affirm the inauspicious association between bronchiectasis and COPD [11], [12], which has recently promoted the revival of the concept of clinical phenotypes in COPD [20]. In this respect, it is important to note that the presence of bronchiectasis is increasingly recognized to be associated with a poorer overall prognosis among COPD patients, as very recently demonstrated by Martinez-Garcia and colleagues [21]. In addition, the fact that bronchiectasis-associated NTM infections have been increasing over the study period, supports a recent US study, which found that subjects with bronchiectasis were 50 to 75 times more likely to have pulmonary NTM infections than those without [9], and illustrates the indispensable need for mycobacterial surveillance including periodical sputum cultures. Moreover, this calls for caution regarding the broad and inadvertent chronic use of macrolide antibiotics as an adjunct immunomodulatory treatment for bronchiectasis and COPD [22]–[24], which may predispose to pulmonary NTM infection [25]. Lastly, our study confirms the diversity of bronchiectasis-associated conditions in the hospitalized general German population. A recent study of 165 bronchiectasis patients identified a specific underlying condition in 74% and this affected the management in 37% of subjects [26]. Hence, an extensive diagnostic work-up as well as frequent, individually tailored and center-based follow-up may be warranted in selected patients.

However, our study has some inherent limitations. First and foremost, the diagnosis of bronchiectasis did not require definite confirmation by HRCT in our study and, on the other hand, we cannot exclude that bronchiectasis patients may have been misdiagnosed, e.g. as asthma or COPD. ICD codes are primarily used for the reimbursement of health services and only secondarily for epidemiological research. They may be subject to potential sources of errors and lack validation for bronchiectasis [27]. In general, ICD codes are considered to have high specificity, but only moderate sensitivity, thus being prone to an underestimation of disease prevalence [28]. Furthermore, due to the depersonalized nature of the data set, we were unable to account for readmissions, which may have had an impact on hospitalization rates, though a substantial overestimation appears unlikely. We could not assess environmental, geographical or sociodemographic patterns and did not analyze the data stratified for individual federal states or urban and rural areas, mainly due to the potential bias related to regional infrastructural differences of healthcare facilities. Moreover, we were unable to investigate the proportion of idiopathic bronchiectasis, which is considered to be a prevalent etiology of bronchiectasis [26], and some other rare specific underlying conditions (e.g. alpha-1 antitrypsin deficiency or PCD), which are typically encoded as non-specific ICD-10 codes (e.g. E88, other metabolic disorders, or Q34, other congenital malformations of respiratory system). The fact that bronchiectasis was the primary diagnosis in only one-fifth of hospitalizations, as previously observed in the US study by Seitz and colleagues [8], illustrates that the three-digit ICD-10 code J47 according to the WHO edition and its German modification does not discriminate between stable disease and acute exacerbation as a substantial proportion of patients obviously entered the hospital due to a more acute or severe condition, mainly (exacerbated) COPD as well as pneumonia. However, the clinical relevance of bronchiectasis as a secondary diagnosis is unknown [8]. Finally, it should be mentioned that our results apply to hospitalized populations in comparable settings only. Bronchiectasis is a chronic condition, which usually requires long-term follow-up care in the outpatient setting, where disease prevalence may be different. A recent report on patient-centered care in outpatient respiratory medicine, which was based upon the billing data of 30 representative German private respiratory practices in 2010, demonstrated that all included practices diagnosed bronchiectasis (ICD-10 J47). Bronchiectasis accounted for on average 0.94% of outpatient diagnoses and correspondingly estimated 30,000 associated consultations in 2010 [29]. In addition, the finding that the majority of bronchiectasis patients in our study died outside the hospital further indicates that the major burden of disease is managed out-of-hospital in Germany. This observation is supported by a recent study from the US, which used national social insurance outpatient claims between 2000 and 2007 and found that the average annual prevalence of 138 bronchiectasis cases per 100,000 population was considerably higher than the rate of 16.5 bronchiectasis-associated hospitalizations per 100,000 population, which the authors had determined previously [8], [9]. Therefore, our data of bronchiectasis-associated hospitalizations are likely to underestimate the overall burden of bronchiectasis. However, as data on the burden of bronchiectasis are limited, our results are an acceptable and the best available surrogate for epidemiological trends across Germany.

The present study is a nationally representative and up-to-date population-based analysis of the burden and trends among almost 62,000 bronchiectasis-associated hospitalizations, including >125 million hospitalizations from 80% of all German hospitals over a 7-year period. To our best knowledge, this is the first analysis of such kind in Central Europe.

### Conclusions

The epidemiology of bronchiectasis is changing, with COPD currently being the most frequent bronchiectasis-associated condition among hospitalized subjects in Germany. Our findings suggest that healthcare facilities, which are managing bronchiectasis patients, need to be able to provide highly specialized care with regard to the complex etiological diagnosis (e.g. CF, immunodeficiencies) and the management of comorbidities and complications against the background of an aging society (e.g. cardiovascular, metabolic, respiratory failure, hemoptysis). Considering the clinical heterogeneity and, in particular, the rarity of some bronchiectasis-associated conditions, prospective national and, preferably, international patient registries, similar to those already existing for CF [30], are needed to further investigate the exact prevalence of bronchiectasis according to the specific underlying condition.
